# Reproductive Ecology of the Lesser Rhea (*Rhea pennata*) in the Peruvian Andes: Nesting Sites, Phenology, and Social Organization

**DOI:** 10.3390/ani16182843

**Published:** 2026-09-09

**Authors:** David E. Samata-Flores, Daniel P. Cáceres Apaza, Mauricio Soto-Gamboa, Juan C. Marin Contreras

**Affiliations:** 1 Laboratorio de Genómica y Biodiversidad, Departamento de Ciencias Básicas, Facultad de Ciencias, Universidad del Bío-Bío, Chillán 3810189, Chile; 2 Facultad de Ciencias Forestales, Universidad Nacional Agraria La Molina, Lima 15024, Peru; 3 Consultoría y Monitoreo Perú S.A.C., Arequipa 04004, Peru; 4 Museo de Historia Natural, Universidad Nacional de San Agustín de Arequipa, Av. Daniel Alcides Carrión s/n, Arequipa 04002, Peru; dcaceresapaza@gmail.com; 5 Laboratorio de Ecología Conductual y Conservación, Facultad de Ciencias, Universidad Austral de Chile, Valdivia 5090000, Chile; msotogamboa@gmail.com

**Keywords:** reproductive phenology, nest monitoring, high-Andean ecosystems, anthropogenic disturbances, ratite conservation

## Abstract

The suri, or lesser rhea (*Rhea pennata*), is a flightless Andean bird facing multiple anthropogenic threats. Despite these pressures, its reproductive ecology remains poorly documented near its distributional limits. This study provides the first comprehensive evaluation of the breeding ecology, nesting distribution, and threats for wild lesser rhea populations at their northern geographic limit in Moquegua, Peru. Across four consecutive breeding seasons (2021–2024), we combined systematic surveys, kernel density analysis, camera trapping, and micro-behavioral nest monitoring. We identified 14 nesting sites comprising 193 nests: 18 old with eggshells remains, 110 old without eggshells, 53 nests under construction, and 12 actives. Kernel density analysis indicated a total nesting area of 195.8 km^2^, with a low density of 0.15 nests/km^2^. Nests were primarily established on moderately sloping hillsides utilizing high-Andean desert vegetation for camouflage. The reproductive period extended from mid-July to mid-December, displaying marked interannual variation in breeding phenology. Camera trap records and field encounters confirmed nest predation and interactions by a native predator guild composed of mammals (*Lycalopex culpaeus*, *Puma concolor*) and raptors, while free-ranging domestic dogs act as localized anthropogenic disturbance factors and linear infrastructure represents potential movement constraints. These findings establish an essential ecological baseline, highlighting that the long-term persistence of this marginal population depends on standardized monitoring to detect subtle demographic shifts and on strategic connectivity conservation across high-Andean landscapes.

## Simple Summary

The lesser rhea (suri), a large flightless bird of the high Andes, is facing an uncertain future, pressed by hunting and the steady loss of the open landscapes it depends on. Yet little is known about how or where it breeds in the wild. Over four consecutive seasons (2021–2024), we followed a remnant population in the highlands of Moquegua, southern Peru. We recorded 14 nesting areas across a mosaic of high-Andean grasslands and shrublands, with nearly 200 nests in total. Breeding extended from mid-July to mid-December, with noticeable year-to-year shifts. Clutches held about 15 eggs on average, and males alone assumed all parental duties, incubating and later leading family groups of up to 18 chicks. Beyond human pressures, the species interacts with natural predators (wild carnivores such as pumas and foxes and raptors such as buzzards and caracaras), while irrigation infrastructure may represent potential barriers to local movement. These findings highlight the high-Andean deserts of southern Peru as a key stronghold for the species, emphasizing the need to safeguard nesting habitats, regulate human activities during the breeding period, and promote conservation alongside local communities.

## 1. Introduction

The lesser rhea (*Rhea pennata*) is a large, flightless bird endemic to South America. Its range extends along the Andes from southern Peru, through western Bolivia, northern Chile and Argentina, reaching the Patagonian steppe [1]. Population relationships and classification remain controversial, extending beyond geographic and phenotypic [2]. Actually, three subspecies have been described [3]: the nominate subspecies, *R. p. pennata*, inhabits lowlands up to 2000 m from southern Chile and Argentina to west-central Argentina in Mendoza [1], while *R. p. tarapacensis* and *R. p. garleppi* inhabit the Andean region up to 4500 m from northern Argentina and Chile to western Bolivia and southern Peru [4]. In Peru, *R. p. garleppi* occurs in the southeast in the department of Puno, and *R. p. tarapacensis* in the southwest in the departments of Tacna and Moquegua, above 3800 m [5]. Despite this geographic differentiation, the taxonomic relationships among these populations remain controversial, and it remains unclear whether the two Andean forms represent distinct evolutionary lineages [2]. Such taxonomic uncertainty has important conservation implications, particularly when geographically and morphologically differentiated populations are treated as a single taxonomic unit. In the case of the lesser rhea, the Andean populations commonly referred to as the Suri or Puna Rhea have small and fragmented populations in the Peruvian Puna, yet their conservation status may be obscured when they are subsumed within the broader *R. pennata* species concept. Resolving the evolutionary relationships and taxonomic status of these populations is therefore important for accurately assessing their conservation needs and for informing appropriate management actions.

Rheas exhibit a polygamous mating system in which males practice simultaneous polygyny, and females engage in sequential polyandry [6,7,8]. In this system, males select a territory, build a nest, and form harems by separating groups of females from larger social groups for courtship. Each female in the harem mates with the male and lays eggs in the nest, assuming the male the exclusive role of incubating the eggs for 35 to 40 days and caring for the chicks [9]. In *R. p. pennata*, the breeding season begins in August and extends until January [10]. Meanwhile, the Andean *R. p. tarapacensis* and *R. p. garleppi* begin egg-laying in September, with nesting ending in December [5]. Changes in photoperiod and climate may influence the start of the breeding season. At the local level, factors such as food availability, nesting site quality, sex ratio, and population density can also modify the reproductive cycle [11]. Reproductive aspects such as clutch size, incubation period, hatching success, and chick care have been well documented for the subspecies *R. p. pennata* [12,13,14]. In contrast, information on the wild Andean population remains scarce [5].

In Peru, information on the reproductive ecology of the lesser rhea (*Rhea pennata*) remains scarce despite its conservation importance, as populations appear to be declining due to habitat loss, predation, hunting, and egg harvesting [15,16,17]. Although several studies have documented aspects of breeding biology, habitat use, and behavior in southern South America, knowledge of reproductive ecology in the Peruvian Andes remains limited [14,16,18]. This lack of information restricts the development of effective conservation strategies for remnant populations inhabiting the high-Andean Puna. Addressing this knowledge gap is particularly important for understanding the factors that may influence the persistence of remnant populations of *R. pennata* in the high-Andean Puna. Accordingly, we investigated the reproductive ecology of a remnant population in the district of Carumas, southern Peru, by integrating information on nesting-site characteristics, reproductive phenology, social organization, and nest dynamics. Specifically, we aimed to (i) identify and characterize nesting sites within the study area, including their spatial distribution, habitat characteristics, and nest density; (ii) document reproductive phenology, including the timing of courtship, copulation, egg-laying, incubation, and chick rearing across breeding seasons; (iii) describe the social organization and group composition during the breeding season; and (iv) characterize nest attributes, reproductive parameters, and potential nest predation through systematic nest monitoring.

## 2. Methods

**Figure 1:**
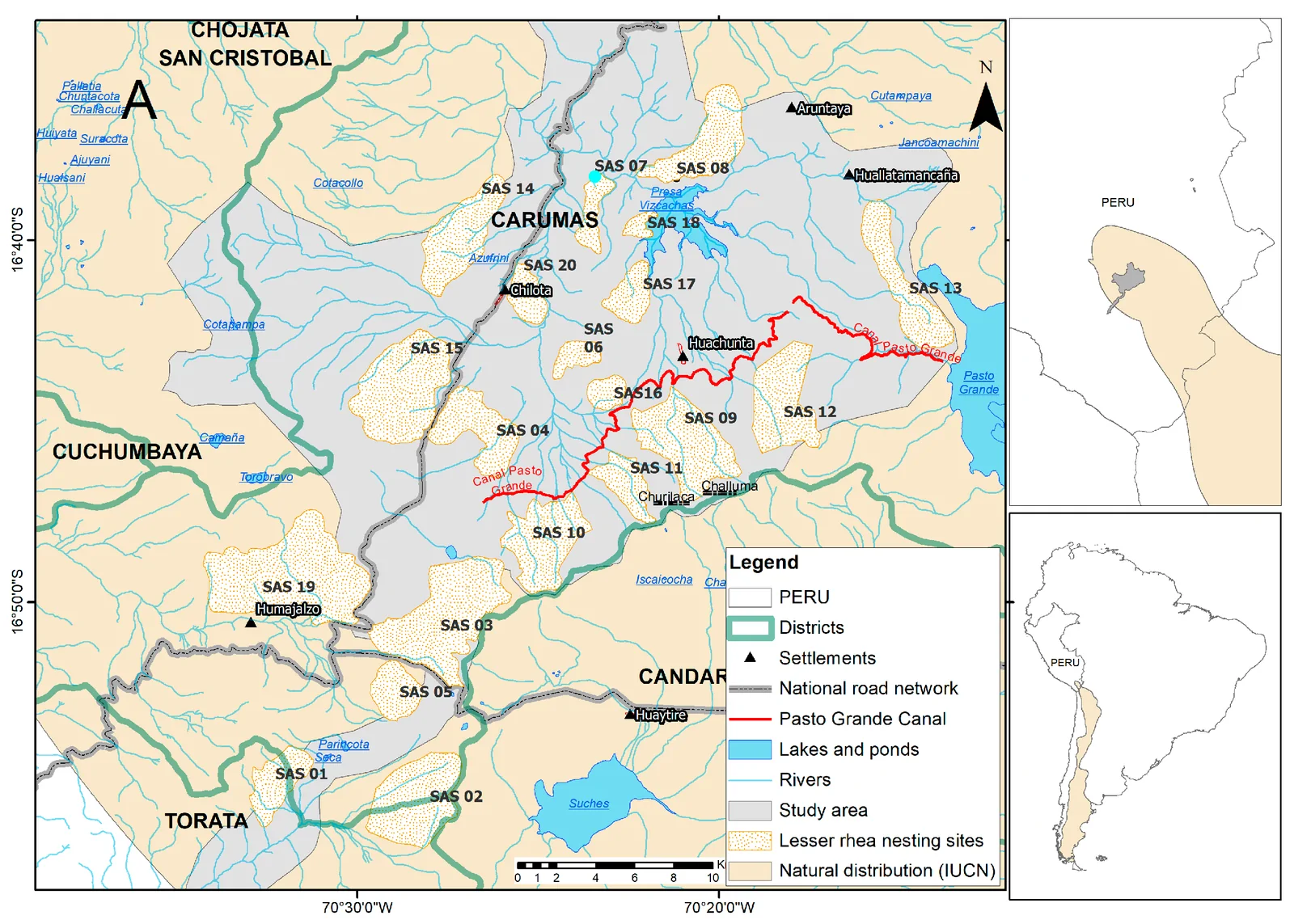

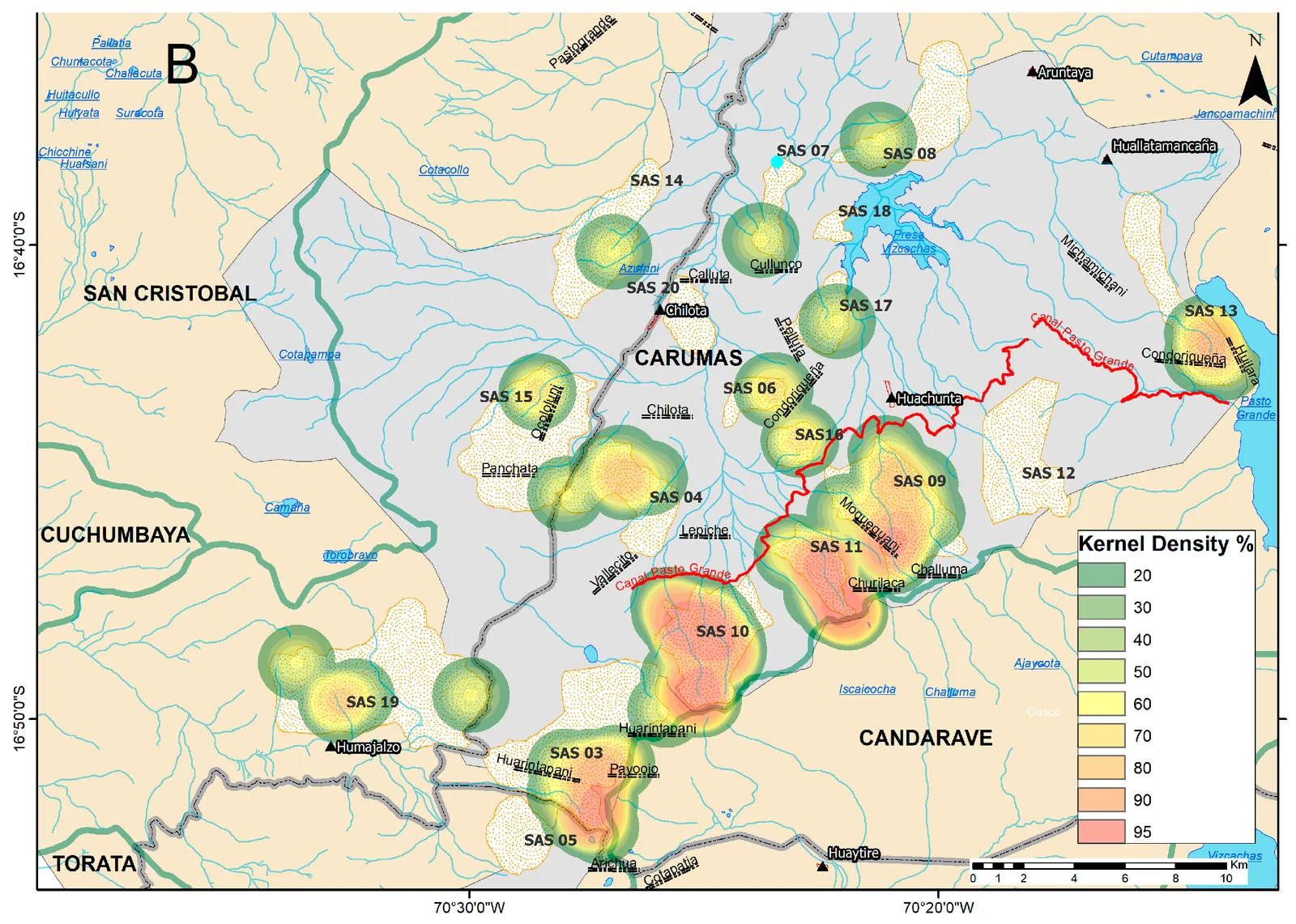
(**A**) Location of 20 identified nesting sites for the Lesser Rhea (*Rhea pennata*) in Mariscal Nieto, Moquegua, Peru. (**B**) Kernel density heat maps of 14 confirmed nesting sites for the Lesser Rhea (*Rhea pennata*) in Mariscal Nieto, Moquegua, Peru.

The study was conducted within the districts of Carumas, San Cristóbal, and Torata, in Mariscal Nieto Province, Moquegua Department, southern Peru (Figure 1A). The study area is located within the high-Andean Puna ecosystem and covers approximately 532 km^2^, with elevations ranging from 4000 to 5000 m a.s.l. It includes several peasant communities and rural annexes, such as Cambrune, Chilota, Huachunta, Aruntaya, Humajalso, Pasto Grande, and Titijones. The landscape is dominated by vegetation typical of the Puna flora, including grasslands, shrublands dominated by *Parastrephia* spp., and *Distichia* sp. peat bogs (Andean high-altitude wetlands). These habitats are interspersed with extensive cold and arid deserts characterized by sparse vegetation adapted to sandy soils of volcanic origin [19,20]. Topographically, the area comprises plains, high-Andean valleys, snow-covered slopes, and mountainous sectors of the Barroso Range, located in the headwaters of the Tambo and Candarave river basins. Fieldwork was conducted between December 2020 and December 2024, covering four breeding seasons (2021–2024). Systematic reproductive observations were carried out primarily between August and December, although general surveys continued throughout the year.

### 2.1. Nesting Site Identification and Spatial Density Analysis

Clutch sites were identified using a targeted, non-probabilistic sampling approach warranted by the low population density and aggregated spatial distribution of the species based on three levels of information: (i) personal communication with residents, (ii) previous records of family groups (adults with chicks), and (iii) records of breeding herds obtained through interviews and preliminary field surveys conducted directly by the authors between 2019 and December 2020. This baseline information defined the Special Surveillance Areas (SAS), which were delineated using natural geographical features (e.g., hills and ravines) and anthropogenic structures (e.g., roads, bridges, and highways). Each SAS was surveyed throughout the reproductive period with a minimum frequency of one weekly visit across four months (between August and November). Reproductive sites were considered confirmed only when at least one physical nest was recorded in the field; for unmonitored areas or those with restricted access, physical nest presence was neither extrapolated nor inferred.

Spatial distribution and reproductive core zones were evaluated using Kernel Density Estimation (KDE) in ArcGIS (Spatial Analyst toolset, Planar method) [21]. To accurately capture the fine-scale spatial clustering of egg-laying sites within each SAS without over-smoothing, a search radius (bandwidth) of 1000 m (1 km) was applied. Density isopleths (50–95% volume contours) were generated to delineate primary breeding zones. Relative nest density was calculated at two distinct spatial scales: at the SAS level, estimated as the ratio between the cumulative total number of nests recorded over the four-year study period (including active nests, nests under construction, nests with eggshell remains, and intact empty nests without eggshells) and the corresponding KDE-derived area; and at the overall study area level, computed as the ratio between the total cumulative KDE surface area and only confirmed active nests and nests with eggshell remains. To prevent spatial estimation bias over non-suitable habitat, a spatial extraction mask was applied to strictly clip and exclude permanent open water bodies. Topographic continuity along steep mountain slopes and high-altitude ridges was maintained, as *Rhea pennata* actively utilizes these high-elevation terrains for egg deposition and parental care.

### 2.2. Social Groups and Reproductive Phenology

Systematic observations were conducted to characterize social structure and reproductive phenology. During each survey, the number of solitary males, groups of females, reproductive groups (one male with several females), juvenile groups, and, at the end of the breeding season, family groups (males with chicks) was recorded. Individuals were classified as adult males, adult females, or juveniles based on body size and behavior [14]. After the reproductive period, mixed groups composed of males, females, and juveniles were also documented. This evaluation was carried out in each SAS at least 5 days a month, in the morning between 07:00 and 13:00.

Reproductive phenology was assessed through direct observations of courtship, copulation, egg-laying, incubation, and chick care. These events were recorded during weekly visits to each SAS throughout the breeding season. During each visit, behavioral states were recorded using instantaneous scan sampling (“snapshots”) at regular intervals, allowing us to document the primary activity of observed individuals and estimate the relative frequency and occurrence of reproductive behaviors without continuous focal tracking.

### 2.3. Nest Search and Characterization

Following the delineation of the SAS, systematic nest searches were conducted on foot along sweep transects and observation points with a wide panoramic view of the site, following a fixed geographic sequence among SAS. Although initial and final transect points remained fixed and overall search effort was kept similar across visits, internal routes were dynamically adjusted by following fresh tracks to ensure complete coverage of the entire area. Observations were made using binoculars (10 × 42) and spotting scopes (Crossfire HD 20–60 × 80 mm, Vortex Optics, Barneveld, WI, USA) from strategic vantage points to minimize disturbance to the birds [14]. Once the nests were detected, we approached them slowly and with caution to reduce the risk of abandonment or increased predation on nests [22]. Each nest was georeferenced using a Garmin eTrex 20 GPS (Olathe, KS, USA) and photographed to describe the environmental characteristics. Nest attributes such as surrounding vegetation, slope, substrate type, and construction material were recorded. Nest diameter and depth were measured when possible, and each nest was assigned an alphanumeric code corresponding to the year of assessment. Nests were classified into four categories following Sarasqueta [12]: (i) old nests with shells, indicating hatched or depredated nests from previous seasons with eggshell fragments incorporated into the nest material; (ii) old nests without shells, inactive structures lacking eggshell remains; (iii) active nests, containing eggs at the time of recording; and (iv) nests under construction, partially built structures with or without covering material, including alternative nests.

For active structures, clutch color, clutch size, and surface temperature were recorded when the incubating male was temporarily absent. Thermal measurements were taken on the unexposed, shaded equatorial region of individual eggs using an infrared thermometer (Ft-888-Genieka, Lima, Peru) to prevent direct solar radiation bias. For abandoned specimens located outside the lay sites, morphometric dimensions (length and width) were measured using a Vernier caliper (Truper S.A. de C.V., Jilotepec, Mexico).

Monitoring intensity varied across the active nests detected during the study. Following a precautionary approach to minimize anthropogenic disturbance, physical intervention, and the risk of nest abandonment by the incubating male, continuous monitoring was not applied uniformly: some active nests were observed exclusively from a distance using binoculars and spotting scopes, while a subset was monitored using camera traps or micro-cameras. Male parental behavior, including incubation duration and nest attendance, was documented using micro-cameras (Mini 550 resolution button screw micro-camera, Stuntcams, Ada, MI, USA) deployed at active nests, with uninterrupted video tracking from laying to hatching achieved for two nests (*n* = 2). To further minimize visual impact, the main body of each camera was buried in the substrate outside the nest cup, leaving only the lens exposed. The lens was discreetly attached to adjacent shrub vegetation surrounding the nest at less than 1 m (<1 m) from the clutch and a height of approximately 10 cm above ground level, pointing directly at the nest bowl. Cameras were installed during early morning hours and operated continuously between 07:00 and 17:00 h.

### 2.4. Camera Trap Monitoring

Camera traps (Bushnell Core S-4K No Glow, Overland Park, KS, USA) were deployed across key locations associated with high reproductive activity, including courtship areas, bofedales, and transit corridors used by conspecifics and laying females. Additionally, these devices were placed near active clutch sites to record male incubation routines, hatching events, and clutch predation. Data obtained from remote phototrapping also served to characterize the overall reproductive phenology of the species, capturing agonistic displays, courtship behaviors, egg-laying activity, and chick emergence across the reproductive season.

Cameras were mounted 40–50 cm above ground level on local vegetation or natural supports, oriented north or south to avoid direct solar glare, and camouflaged with surrounding natural vegetation to minimize visual disturbance. For active nests, cameras were positioned 10–20 m from the nest bowl. To minimize human disturbance during the incubation phase and reduce the risk of nest abandonment, cameras at active nests were installed in a single intervention upon nest detection and left undisturbed for the remainder of the breeding period.

Overall, field sampling across the five-year study period (2020–2024) accumulated 224 field days, 1120 h of direct observation, and 1530 km of walked transects, alongside 7380 camera-trap days and close-range video monitoring using two micro-cameras (*N* = 2) at selected active nests exclusively during the 2021 breeding season. The camera-trap stations were established and maintained across the four monitoring years (2021–2024) to ensure consistent multi-year sampling. Table 1 summarizes a detailed annual breakdown of sampling effort and field monitoring coverage.

Sampling in 2020 corresponded to an initial survey focused on SAS delimitation and family group counts during the post-breeding period. From 2021 to 2024, evaluations covered four complete breeding periods, combining systematic search transects, social group and reproductive phenology evaluations, and camera-trap surveys.

Given the descriptive nature of the study, continuous variables were summarized as means, standard deviations, medians, and ranges. Inferential analyses were applied where sample sizes were sufficient, including chi-square (*χ*^2^) tests to evaluate habitat preferences and Spearman rank correlations (*ρ*) for spatial relationships. Small sample sizes restricted interannual statistical modeling for specific reproductive parameters. Video footage was analyzed using VLC media player (version 3.0.17.4), while kernel density analyses and map production were performed using Google Earth Pro (version 7.3.6, Google LLC, Mountain View, CA, USA) and ArcGIS (version 10.5, ESRI, Redlands, CA, USA).

## 3. Results

### 3.1. Nesting Sites

**Figure 2:**
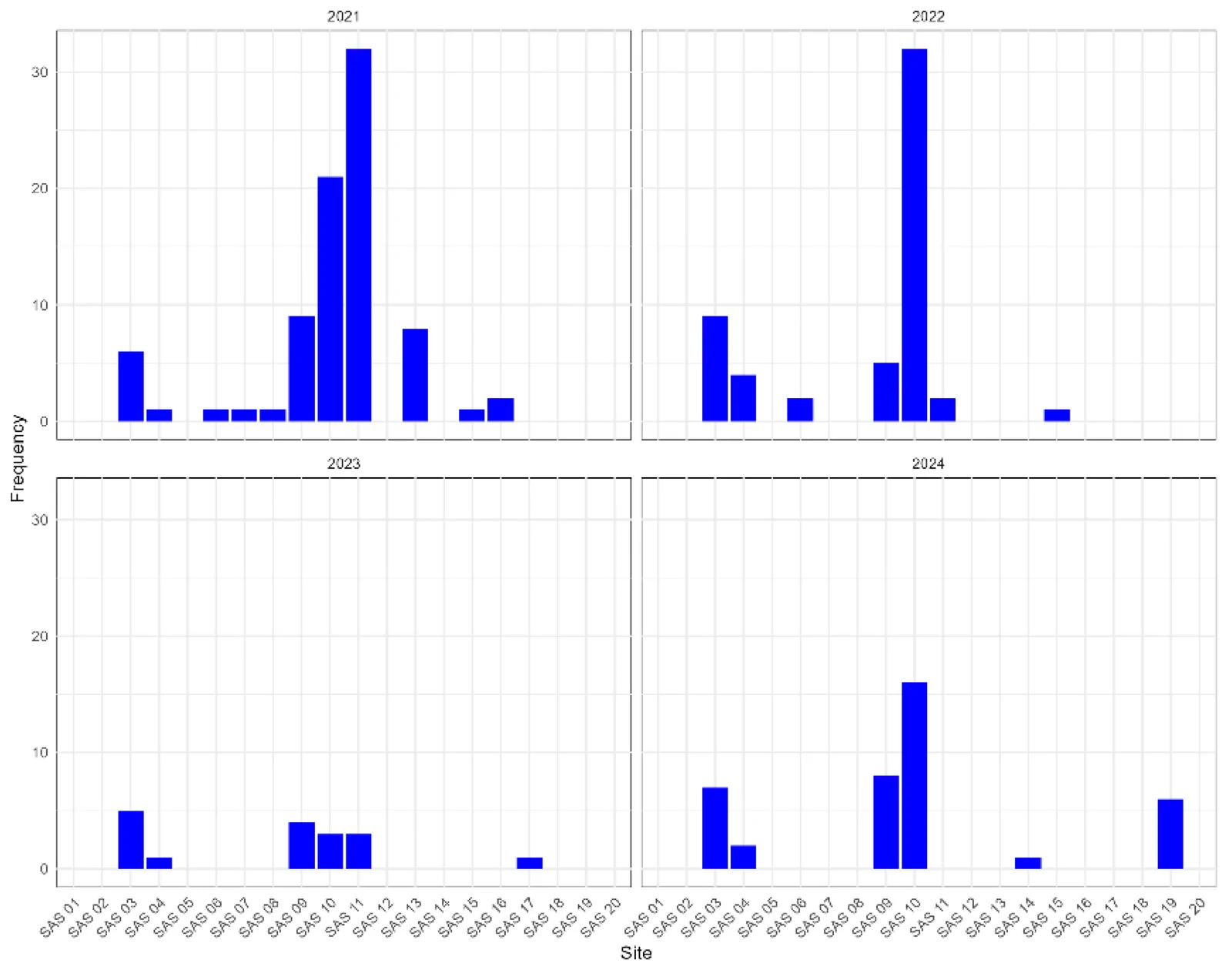
Total number of nests found at the evaluated sites during the 2021–2024 breeding seasons.

Fourteen confirmed nesting sites were identified among the 20 proposed Special Areas for Surveillance (SAS), comprising a total of 193 rhea nests (Figure 1B and Figure 2). The 95% kernel density analysis delineated an effective nesting area of 195.8 km^2^ and revealed a heterogeneous spatial distribution, with higher nest concentrations in SAS 10, SAS 11, SAS 3, SAS 9, and SAS 13. Recurrent laying activity was recorded in SAS 3 (Arichua Payoojo), SAS 9 (Vizcachane–Achacala), SAS 10 (Challahuichinca), and SAS 11 (Lipichi), whereas SAS 13 (Pasto Grande–Huilara) was in a more isolated sector.

The highest nest density was recorded in SAS 10 (Challahuichinca), with 72 nests (2.84 nests/km^2^), followed by SAS 11 (Lipichi), with 37 nests (2.07 nests/km^2^; Figure 1B and Figure 2, Table 2). Direct field evaluations could not be performed in certain SAS (SAS 1, SAS 2, SAS 5, SAS 12, and SAS 20) due to access restrictions associated with private property and mining concessions. However, systematic observation of young chicks (*charitos*) led by adult males in adjacent or surrounding areas confirms the functional use of these sites as rearing habitat. It provides indirect evidence of active nesting nearby.

### 3.2. Reproductive Phenology and Social Groups

**Figure 3:**
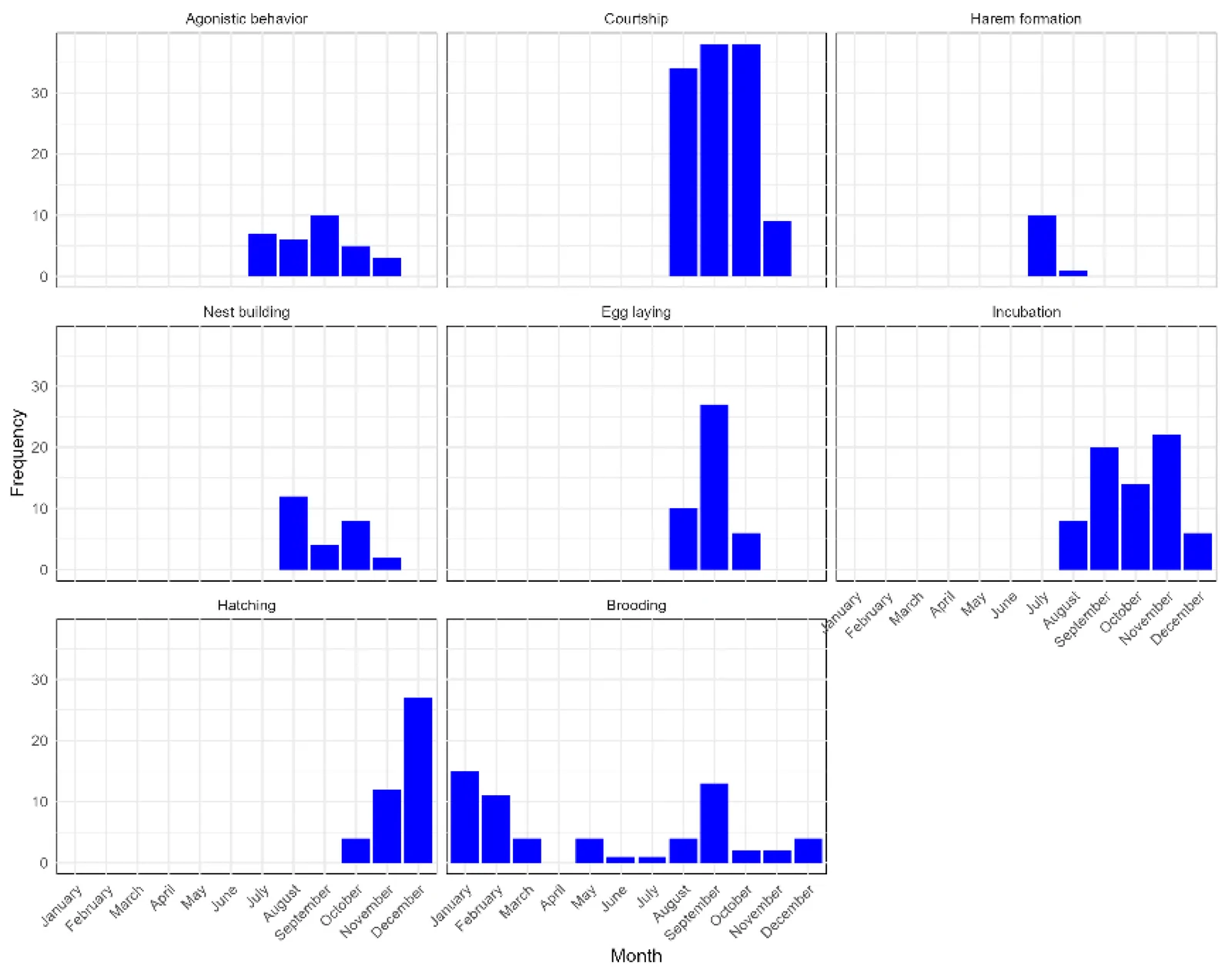
Histogram of the frequency of occurrence of reproductive behaviors over time on a monthly scale.

Reproductive phenology was determined based on 755 direct observations and camera-trap records collected between December 2020 and December 2024. Two main periods were distinguished: a non-reproductive period from January to June and a reproductive period extending from mid-June to mid-December, with minor interannual variation (Figure 3). In 2023, an unusual record of reproductive activity was documented as early as June.

During the non-reproductive period, individuals were observed in mixed herds, family groups (adult males with chicks or juveniles), groups of adults that had completed reproduction, and juvenile-only herds. No agonistic interactions or harem formation were recorded during this phase.

**Figure 4:**
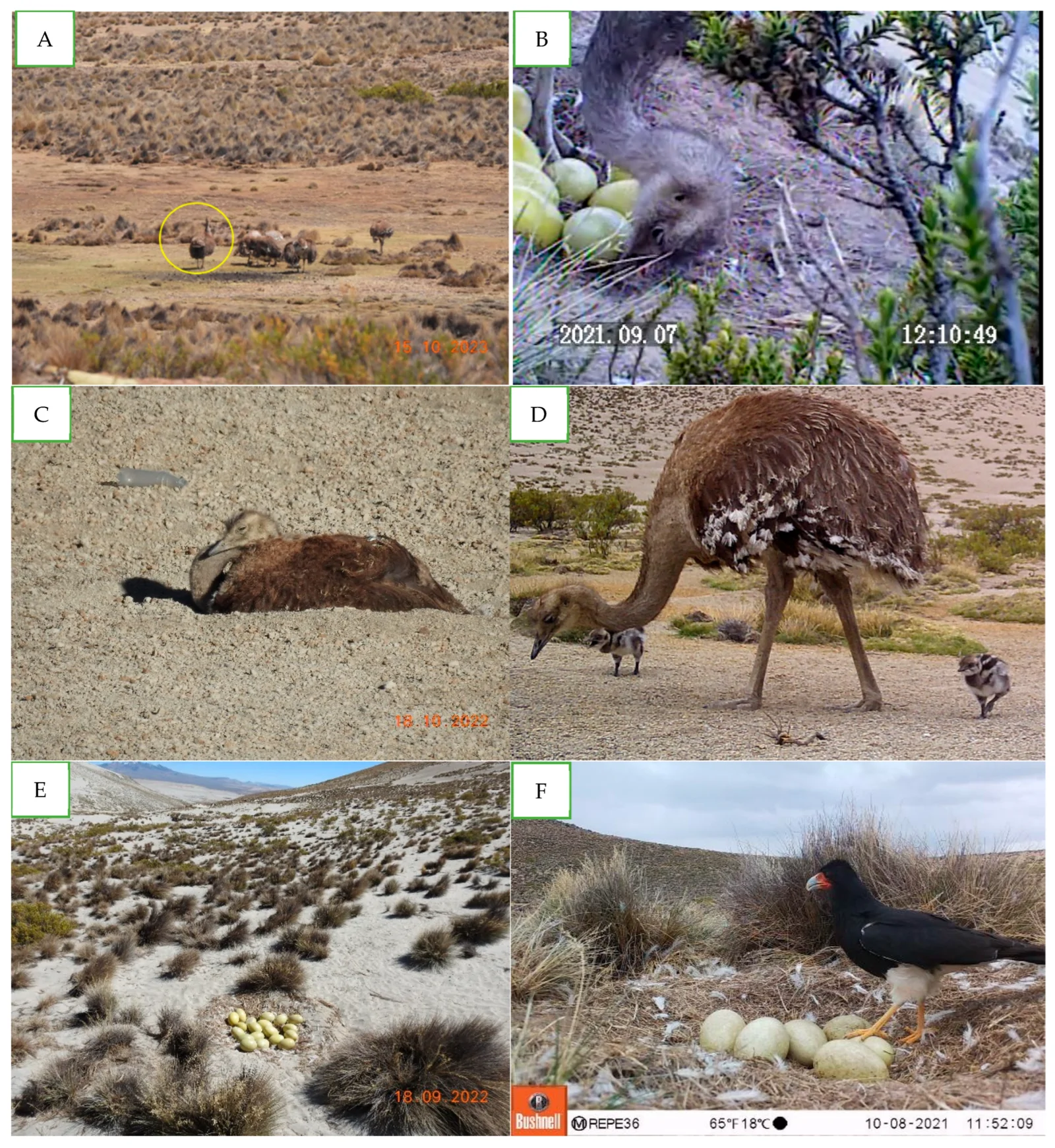
Field photographs (**A**) Reproductive harem in courtship; the adult male leading the group is highlighted within the yellow circle. (**B**) Removal of eggs from nest recorded by camera trap. (**C**) Incubation in high Andean desert. (**D**) Parental care of rhea chicks. (**E**) *Rhea pennata* nest on a hillside. (**F**) *Daptrius megalopterus* preying on eggs in an unattended nest. Photos by the authors.

The reproductive period followed five sequential stages: pre-laying, egg-laying, incubation, hatching, and chick-rearing [23]. The pre-laying stage, occurring mainly between late June and October, was characterized by agonistic interactions among males, courtship displays, copulation, and nest construction. We recorded 24 agonistic encounters between males, primarily involving aggressive posture displays, wing-flapping, vocalizations, and short territorial chases to establish dominance and defend access to females. Courtship behavior was observed from late July to mid-November, with 153 courtship events and six copulations documented, mostly between mid-July and mid-October (Figure 4A).

Egg-laying occurred between the last week of August and the third week of October. Among 29 monitored nests, 7% initiated laying in August, 48% in September, and 45% in October. The egg-laying period lasted 7–10 days (mean = 8.5 ± 1.2 days; *n* = 6), with most egg-laying events occurring during the afternoon (13:00–17:00 h). The incubation period extended from early September to mid-December and lasted an average of 43.5 days (*n* = 2 nests monitored from laying to hatching; Figure 4B,C).

Hatching occurred between mid-October and mid-December. Chicks became mobile within three days of hatching and followed the male, which provided exclusive parental care during the brood period, extending from hatching until June of the following year (Figure 4D). Juveniles remained with the male for up to seven months, and in some cases more than 12 months. A total of 48 family groups and 357 chicks were recorded from 2020 to 2024 (Table 3). Both the number of family groups and total chick abundance increased progressively, peaking in 2023 (18 groups, 146 chicks) and reaching a minimum in 2021 (5 groups, 36 chicks). The mean number of chicks per family group ranged from 6.3 (2022) to 10.3 (2020), with group sizes varying from a minimum of 1 chick (2023 and 2024) to a maximum of 18 chicks (2020 and 2024). Additionally, the date of the first chick sighting advanced notably over the years, shifting from early December in 7 December 2020 to mid-November in 2021 and 2022 and reaching mid-October in the 2023 and 2024 seasons.

Social organization during the study included: (i) solitary individuals, mainly males during the reproductive period (130 records); (ii) groups of adult females searching for a male (45 groups); (iii) reproductive harems composed of one male and one to six females (153 records), with an average of 2.54 ± 1.06 females per male; (iv) family groups (broods) consisting of one male with chicks (mean = 7.27 ± 3.8 chicks); (v) juvenile herds independent of the male; (vi) herds of non-reproductive adults; and (vii) mixed herds composed of adults, juveniles, and subadults.

### 3.3. Nest Characterization

A total of 12 active nests with eggs, 18 old nests with eggshell remains, 110 old nests without shells, and 53 nests under construction were recorded. Egg-containing units were most frequent in 2021 (*n* = 6), followed by 2022 (*n* = 3), 2024 (*n* = 2), and 2023 (*n* = 1). Nests consisted of circular ground depressions that deepened and accumulated plant material as construction progressed. Mean structure diameter was 93.7 ± 21.2 cm, and depth was 23.0 ± 8.6 cm (*n* = 139). Depression lining included plant debris from grasslands and shrublands, as well as feathers from the brood patch.

Nest placement differed significantly across habitat types (*χ*^2^ = 278.4, df = 4, *p* < 0.001), demonstrating a strong non-random concentration in mixed grassland–shrubland vegetation (75.6%, *n* = 105), followed by grassland (11.9%, *n* = 16), shrubland (6.7%, *n* = 9), high-Andean desert (3.6%, *n* = 5), and mixed grassland–shrubland–yareta vegetation (2.1%, *n* = 3). Approximately half of the nests were located on moderate slopes (48.2%) and half on gentle slopes (51.8%), all on sandy substrates. The mean distance to the nearest water body or wetland was 1214 ± 895 m (range: 2–3570 m). Distance to wetlands did not show a linear relationship with slope or nest dimensions (Spearman *ρ* = −0.12, *p* = 0.16).

Clutch size averaged 14.8 ± 4.5 eggs (median = 15.0 eggs, IQR = 6.25, *n* = 8), ranging from 5 to 19 eggs, with an estimated laying rate of 5.1 ± 0.6 eggs per female per nest. Given the small number of active nests per breeding season (*n* = 1–6 nests/year), formal parametric interannual comparisons (e.g., ANOVA) and environmental regression models for clutch size were not statistically robust due to high risk of type II errors and parameter overfitting; thus, parameters are presented as empirical baseline reference data. Eggs were oval, measuring 128.1 ± 10.1 mm in length and 87.2 ± 11.8 mm in width (*n* = 19), with an average mass of 499 ± 38.5 g (*n* = 6). Egg coloration changed from bright greenish-yellow to dull yellow or whitish as incubation progressed (Figure 4E and Figure 4F, respectively). Mean egg temperature during incubation was 33.5 ± 1.2 ^∘^C (*n* = 19). Hatching success in two continuously monitored nests was 71%.

Incubation behavior monitored in two nests during the early days of incubation revealed the behavioral categories described by Bruning [24], including nest maintenance, vigilance, resting, and foraging. Nest maintenance activities occurred throughout the day and included preening, egg shifting, position rotation, egg retrieval, and material collection. Preening and ventral feather-plucking events lasted 60–70 s and occurred both while crouching and standing, causing abundant belly feathers to fall onto the eggs. Egg shifting involved minor incubation interruptions where the male moved 1–3 eggs with its bill while leaning on its tarsi without rising, occurring 3–7 times per day with an average duration of 25 s (Figure 4B). Nest rotation (spatial reorientation) occurred primarily from midday onward (starting at approximately 11:00 h), lasting about 5 s per turn. Egg retrieval, consisting of gently pulling newly laid eggs from the nest periphery into the central clutch with the bill, took approximately 5 s after each laying event. Material collection and sanding, performed by extending the neck backward to gather plant debris and sand, occurred throughout the day with event durations ranging from 1 to 5 min.

Regarding the diurnal time budget, vigilance accounted for 25% of the total time, manifested as both alert crouching (eyes open, neck slightly extended) and standing vigilance over the nest. Resting occupied 18% of the diurnal incubation time and was recorded intermittently between 13:00 h and 18:00 h, characterized by closed eyes, somnolence, and the neck retracted into an ‘S’ shape over the back. Foraging represented approximately 11% of the diurnal budget and involved two distinct strategies: consuming surrounding vegetation without rising and active off-nest foraging trips in nearby areas. These off-nest trips occurred once a day during the morning and midday, with an average duration of 20 min and a maximum departure of 126 min. Notably, during a snowfall event that began in the late afternoon of October 5 and persisted through the early morning of October 6—melting completely by 10:00 h—the attending male remained strictly on the nest without rising, prioritizing clutch thermoregulation and shielding the eggs from snow accumulation.

### 3.4. Predation and Nest Damage

Camera-trap monitoring across 10 active nests (300 camera-days) revealed that nest predation was predominantly driven by nocturnal mammalian carnivores—mainly the culpeo fox (*Lycalopex culpaeus*)—and diurnal avian predators such as the Andean caracara (*Daptrius megalopterus*; Figure 4F), with minor contributions from domestic dogs, variable hawks, and pumas (Table 4). Additionally, physical nest damage was primarily caused by ungulate trampling, dominated by wild vicuñas (*Vicugna vicugna*) and, to a lesser extent, by domestic livestock and tarucas (Table 4). Puma (*Puma concolor*) activity in the nesting area included a confirmed lethal attack on an active nest, where a puma killed the incubating adult rhea and depredated the eggs—evidenced by fresh attack marks on the carcass, tracks, and total nest destruction. Additionally, camera traps yielded 8 independent records of pumas prowling near active nesting sites.

No re-nesting attempts or secondary clutches were observed following nest destruction or abandonment. Although adult males were sporadically recorded visiting destroyed or degraded nest sites, it was not possible to confirm individual identity or determine whether these visits represented re-nesting motivation or non-functional inspection behavior.

## 4. Discussion

This study provides the first comprehensive description of nesting distribution, reproductive phenology, social organization, and factors influencing nesting success for wild populations of the Lesser Rhea (*Rhea pennata*) at the northern limit of its distribution in southern Peru. By identifying 14 nesting sites, characterizing reproductive timing and nesting ecology over consecutive breeding seasons, and evaluating key factors influencing nest outcomes, our results establish a critical baseline for understanding population dynamics in a marginal Andean environment. Identifying these fourteen nesting sites in Moquegua represents a substantial expansion of the known nesting range of *R. pennata* in Peru. Previous studies reported egg-laying zones restricted to Tacna [5] and Puno [25], while potential clutch placement near Laguna Toro Bravo, near Humajalso, had only been weakly suggested [26]. Our findings confirm and extend these observations by documenting active nesting in areas such as Humajalso, Chilota, and other isolated high Andean desert localities where nesting had not been previously evaluated. In addition, kernel density analysis indicated a total breeding landscape of 195.8 km^2^, with a nest density of 0.15 nests/km^2^. Although this value appears quantitatively similar to the average density of 0.17 nests/km^2^ reported for southern populations of *R. pennata* in northwestern Patagonia [14], these estimates stem from differing methodological approaches and analytical frameworks and should therefore be compared cautiously as descriptive trends. Nevertheless, this pattern aligns with the biogeographical hypothesis that northern high-Andean populations tend to occur at lower densities near their distributional limits [27,28], where harsher climatic conditions, fragmented habitats, and heightened anthropogenic pressures may constrain population size and reproductive output [29].

The selection of clutch locations also reflects local ecological adaptations to these extreme environments. Laying depressions were primarily located on moderately sloping hillsides, often in remote sectors with mixed grassland and shrub vegetation that provides critical visual camouflage against natural enemies. Interestingly, the use of sparsely vegetated or barren high Andean desert terrain, often more than 1 km away from bofedales, contrasts directly with patterns reported for southern conspecifics that preferentially deposit eggs closer to wetland edges [10]. This flexibility in microhabitat selection may reflect local trade-offs between avoidance of carnivores and raptors, human disturbance, and access to foraging areas. Despite these adaptations, linear infrastructure may act as physical or ecological barriers in the study area. In this context, the Pasto Grande Canal could restrict movement between nesting habitats and key foraging wetlands. During field surveys, female groups were observed on four occasions walking parallel to the canal bank opposite an isolated male, suggesting potential intent to cross. Furthermore, two fatal drowning incidents of adult suris were recorded in the canal during the study period. While individual movement patterns and crossing behaviors were not systematically tracked, these direct field observations suggest that such infrastructure could increase energetic costs and mortality risks during daily movements. Similar infrastructure-driven fragmentation has been shown to affect movement and reproductive success in other large ground-dwelling birds [30], highlighting the importance of evaluating landscape connectivity in future regional management plans.

Regarding reproductive timing, our results show that the reproductive period of *R. pennata* in Moquegua extends from mid-July to mid-December, while the non-reproductive period spans January to June. This timing broadly overlaps with that reported for wild populations in northern Chile [31] and differs substantially from southern Patagonian populations, where reproductive activities regularly begin earlier in the year [32]. These macro-ecological differences likely reflect latitudinal and altitudinal gradients in photoperiod, temperature, and primary productivity, which are known to dictate avian reproductive timing [33,34]. Nevertheless, rather than strict calendar dates, our data emphasize a marked interannual variability in reproductive events, including egg laying, hatching, and chick emergence. Such variability suggests a highly flexible reproductive strategy, allowing individuals to adjust breeding investment in response to immediate local environmental conditions, including unpredictable food availability, sudden flooding of nesting sites, and fluctuating predation pressure. This flexibility in reproductive strategies has been proposed as a key mechanism enabling the persistence of large ratites in highly variable environments [35]. It may be particularly vital for the survival of the marginal populations studied here. This strategy is also reflected in the reproductive investment; while the mean clutch size recorded (14.8 ± 4.5 eggs) falls well within the range reported for other Andean populations [32], the total number of active nests recorded over four breeding seasons was remarkably low compared to southern populations [31]. Together, these results suggest that the reduced population density observed in northern Peru is driven more by fewer breeding attempts than by reduced per-nest reproductive investment.

The interannual variations in the number of family groups and total chicks from 2020 to 2024, alongside shifting dates of the first chick sightings, must be interpreted with caution, as they are likely influenced by differences in annual sampling effort. In long-term monitoring of cryptic, low-density ratites, variations in field logistics and survey intensity can significantly alter detection probabilities, thereby inflating or deflating annual counts and distorting phenological trends. This methodological constraint is particularly evident in the delayed baseline recorded during the first year; the late date of the first chick sighting in 7 December 2020 is primarily attributed to the fact that field monitoring that season only commenced in December, which naturally truncated the detection of earlier hatching events that were successfully documented in mid-October during subsequent, more intensely surveyed years such as 2023 and 2024. Consequently, the lower reproductive output recorded in 2020 (4 groups, 41 chicks) and 2021 (5 groups, 36 chicks) likely represents a sampling artifact rather than a biological shift. Despite this, the dynamic nature of the social structure observed during the breeding season—consisting of solitary males, groups of females, reproductive harems, and post-breeding family groups—largely corresponds with descriptions for other *Rhea* species [12,23]. The presence of large family groups, with up to 18 chicks per single male, confirms the central role of exclusive male parental care and strongly supports chick adoption [14,36], a cooperative behavior previously documented in rheas and other ratites [14,32]. The prolonged association of juveniles with the male, extending up to or exceeding 12 months, underscores the high dependency of offspring on paternal guidance in these harsh environments. From a conservation monitoring perspective, this complex social system, combined with high post-hatching mobility, complicates the precise field estimation of reproductive success and juvenile survival because chick adoption obscures genetic parentage and individual fate, highlighting the need for long-term monitoring approaches that integrate demographic, behavioral, genetic, and spatial data.

At a finer scale, early incubation monitoring validated the ethological categories described by Bruning [24], revealing crucial diurnal time allocation and nest maintenance strategies. Behaviors such as ventral feather-plucking, egg turning, and rapid retrieval of peripheral eggs function as vital mechanisms for efficient embryonic thermoregulation and clutch integrity. Diurnally, the brooding male balanced high vigilance with intermittent rest and brief foraging trips, optimizing both clutch defense and energetic maintenance. Notably, during a recorded snowfall, the attending male remained strictly on the nest, prioritizing egg thermoregulation over self-maintenance. This remarkable behavioral commitment underscores the substantial energetic costs and resilience of males during early incubation in harsh high-Andean environments, where parental investment is constantly challenged by severe natural and anthropogenic pressures. Camera trap records and direct field observations documented active nest interactions and predation by a native predator guild composed of both mammals and raptors, including the culpeo fox (*Lycalopex culpaeus*), Andean caracara (*Daptrius megalopterus*), and variable hawk (*Geranoaetus polyosoma*), aligning with species interactions reported across the high Andes and Patagonia [11,37]. Notably, regarding apex predators, an event of adult *Rhea pennata* predation by a puma (*Puma concolor*) was directly recorded during fieldwork. Furthermore, camera traps and sign encounters confirmed concurrent and frequent use of sectors with the highest nest concentrations. Beyond native fauna, free-ranging domestic dogs (*Canis familiaris*) associated with local livestock grazing were repeatedly recorded during systematic search transects, particularly within feeding grounds used by suris during the courtship and pre-nesting period. Rather than direct egg predation, the persistent presence of dogs in these foraging areas represents a significant anthropogenic disturbance that disrupts natural courtship displays, increases vigilance behavior, and induces spatial displacement during a critical phase of the breeding cycle. Such recurring human-associated disturbances near key foraging and nesting grounds constitute chronic stressors that may impair long-term reproductive recruitment in this vulnerable high-altitude population [38]. Finally, we acknowledge that this study is primarily descriptive and was constrained by limited sample sizes for some reproductive and behavioral parameters, particularly regarding precise hatching success, juvenile chick survival, and the small number of intensely monitored nests. To address these limitations, future studies should prioritize long-term demographic monitoring, individual marking or satellite telemetry, and rigorous quantitative assessment of habitat connectivity between nesting and feeding wetlands. Expanding behavioral and ecological research to neighboring regions such as Tacna, Puno, northern Chile, and western Bolivia is urgently required; such collaborative efforts would allow for a comprehensive evaluation of regional connectivity, metapopulation dynamics, and population genetic structure at a broader, international scale.

## 5. Conclusions

In conclusion, this study establishes a critical ecological baseline for the northernmost wild populations of the Lesser Rhea (*Rhea pennata*) in Moquegua, Peru, significantly expanding its known geographical nesting range. Our findings show that while these marginal Andean populations exhibit a predictable macro-ecological reproductive phenology aligned with regional photoperiods, they also show high reproductive flexibility and localized nest-site adaptations to cope with extreme high-altitude environments. At a finer scale, the high energetic investment and behavioral commitment displayed by incubating males—particularly during adverse weather events like snowfalls—demonstrate a key adaptive strategy where time and energy spent during incubation enhance reproductive output and offspring survival under extreme environmental conditions. However, the low nest density, combined with localized anthropogenic pressure from free-ranging domestic dogs and potential movement constraints imposed by linear infrastructure, presents ongoing challenges to long-term population recruitment. Furthermore, this study demonstrates that multi-year demographic evaluations of cryptic ratites must systematically account for variations in sampling effort to avoid phenological misinterpretations. Ultimately, conserving this threatened ratite at its northern distributional limit requires transitioning from localized descriptive efforts toward integrated, international monitoring strategies that implement satellite telemetry and genetic tools across Peru, Chile, and Bolivia to ensure regional landscape connectivity and metapopulation persistence.

## Figures and Tables

**Table 1:** Annual sampling effort and field monitoring coverage (2020–2024).

Year	Months Assessed	Total Field Days	Direct Observation Hours	Transect Distance (km)	Habitat/Corridor Camera Stations (*N*)	Active Nest Camera Stations (*N*)	Total Fixed Camera-Trap Stations (*N*)	Camera Trapping Effort (Trap-Days)
2020	December	12	60	46	—	—	—	—
2021	August–December	56	280	392	22	4	26	1560
2022	August–December	54	270	378	21	2	23	1840
2023	August–December	55	275	385	24	2	26	1820
2024	August–December	47	235	329	25	2	27	2160
Total	2020–2024	224	1120	1530	92	10	102	7380

**Table 2:** Summary of the evaluated nesting sites, political location, dominant ecosystem, kernel area, number and density of nests, and important notes.

Site ID	Site Name	Dominant Ecosystem	Kernel Surface (km^2^)	Nests (*n*)	Density (Nests/km^2^)	Observations
SAS 1	Titijones Oeste	Shrublands (*Parastrephia* spp.)	-	-	-	Private property (mining claims); chicks recorded.
SAS 2	Titijones Este	Mixed grassland–shrubland	-	-	-	Private property (mining claims); records of chicks.
SAS 3	Arichua Payoojo	High-Andean desert	27.08	27	0.99	Light livestock activity; annual nesting recorded.
SAS 4	Chilota sand dunes	High-Andean desert	17.42	7	0.40	Heavy livestock activity; includes scrubland/wetlands.
SAS 5	Titijones-Binational	Shrublands (*Parastrephia* spp.)	-	-	-	Adjacent to private property; previous chick records.
SAS 6	Chilota III	Mixed grassland–shrubland	7.04	2	0.28	Constant livestock activity; fenced wetlands.
SAS 7	Pagrapampa-Ingenio	Mixed grassland–shrubland	6.78	1	0.15	Slopes of Collunco/Pelluta; little livestock activity.
SAS 8	Aruntaya	Shrublands (*Parastrephia* spp.)	6.67	1	0.15	Reservoir presence and communal access road.
SAS 9	Vizcachane-Achacala	Mixed grassland–shrubland	27.65	26	0.94	Light livestock; annual nesting; base of Challuma mt.
SAS 10	Challahuichinca	Shrublands (*Parastrephia* spp.)	25.36	72	2.84	Wetland cut by Pasto Grande canal; recurrent nesting.
SAS 11	Lipichi	Grasslands	17.89	37	2.07	Features peat bogs; moderate livestock; recurrent nesting.
SAS 12	Surapatilla-Musiña	Mixed grassland–shrubland	-	-	-	Potential area; breeding harems recorded in 2021.
SAS 13	Pasto Grande-Huilara	Mixed grassland–shrubland	10.76	8	0.74	Rural road presence; sporadic livestock activity.
SAS 14	Kalamarca	Grasslands	6.78	1	0.15	Grasslands/wetlands; constant livestock; vehicular access.
SAS 15	Ocololuni	Shrublands (*Parastrephia* spp.)	7	2	0.29	Near Panchata mountain; moderate livestock activity.
SAS 16	Condoriquiña	Shrublands (*Parastrephia* spp.)	6.92	2	0.29	Southern slope; wetlands with livestock fences.
SAS 17	Sayhuane-Ancoara	Grasslands	6.78	1	0.15	Grassland hills; constant livestock grazing.
SAS 18	Calasaya 1	Shrublands (*Parastrephia* spp.)	-	-	-	Adjacent to Vizcachas Reservoir; chicks present in 2023.
SAS 19	Humajalso-Cambrune	High-Andean desert	21.67	6	0.28	Solar energy projects; significant livestock activity.
SAS 20	Caluta 1-Culco	Grasslands	-	-	-	Private property; reproductive herds recorded.
Total	-	-	195.81	193	-	-

Note: Kernel surface area represents the 95% isopleth.

**Table 3:** Number of family groups, chicks, and date of the first chick sighting in each breeding season.

Reproductive Parameter	2020	2021	2022	2023	2024
Family groups (*n*)	4	5	9	18	12
Total chicks (*n*)	41	36	57	146	77
Chicks per family group (mean)	10.3	7.2	6.3	8.1	6.4
Minimum chicks per group (*n*)	5	2	3	1	1
Maximum chicks per group (*n*)	18	13	13	17	18
Date of first chick sightings	7 December	13 November	12 November	18 October	17 October

Note: Data based on field surveys in Moquegua, Peru. Date formats follow the month-day structure.

**Table 4:** Summary of nest predation and physical nest damage events recorded by camera traps across 10 monitored active nests (2021–2024).

Event Category	Species/Agent	Target Item	Event Count (*n*)	Relative Frequency (%)	Event Rate (Events/100 Camera-Days)
Nest Predation	*Lycalopex culpaeus*	Eggs	38	65.50%	12.67
	*Daptrius megalopterus*	Eggs	16	27.60%	5.33
	*Canis familiaris*	Scavenging/Eggs	2	3.40%	0.67
	*Geranoaetus polyosoma*	Eggs	1	1.70%	0.33
	*Puma concolor*	Eggs/Attending adult	1	1.70%	0.33
Subtotal Predation	-	-	58	100.00%	19.33
Physical Nest Damage	*Vicugna vicugna*	Nest structure/Eggs	17	77.30%	5.67
	Domestic livestock (*Llama*)	Nest structure/Eggs	3	13.60%	1
	*Hippocamelus antisensis*	Nest structure/Eggs	2	9.10%	0.67
Subtotal Damage	-	-	22	100.00%	7.33

## Data Availability

The raw data supporting the conclusions of this article will be made available by the authors on request.
